# Screen use and internet addiction among parents of young children: A nationwide Canadian cross-sectional survey

**DOI:** 10.1371/journal.pone.0257831

**Published:** 2022-01-31

**Authors:** Cindy-Lee Dennis, Sarah Carsley, Sarah Brennenstuhl, Hilary K. Brown, Flavia Marini, Rhonda C. Bell, Ainsley Miller, Saranyah Ravindran, Valerie D’Paiva, Justine Dol, Catherine S. Birken

**Affiliations:** 1 Lawrence S. Bloomberg Faculty of Nursing, University of Toronto, Toronto, Canada; 2 Li Ka Shing Knowledge Institute, St. Michael’s Hospital, Toronto, Canada; 3 Department of Psychiatry, University of Toronto, Toronto, Canada; 4 Public Health Ontario, Toronto, Canada; 5 Dalla Lana School of Public Health, University of Toronto, Toronto, Canada; 6 Interdisciplinary Centre for Health & Society, University of Toronto Scarborough, Toronto, Canada; 7 Department of Agricultural, Food and Nutritional Sciences, University of Alberta, Edmonton, Canada; 8 Lakehead University, Thunder Bay, Canada; 9 York Region Public Health, Vaughan, Canada; 10 Faculty of Medicine, University of Toronto, Toronto, Canada; 11 Hospital for Sick Children, Toronto, Canada; Georgia Institute of Technology, UNITED STATES

## Abstract

**Objectives:**

To establish the factorial structure and internal consistency of the Internet Addiction Test (IAT) in parents, the level and correlates of problematic internet use, and patterns and types of screen use.

**Study design:**

Data were collected through an online questionnaire about preconception health among Canadian women and men with ≥1 child. The questionnaire included the IAT and questions about time spent on screens by device type, use of screens during meals and in the bedroom, and perceptions of overuse. Factor analysis was completed to determine the factorial structure of the IAT, with multivariable linear regression used to determine correlates of the IAT.

**Results:**

The sample included 1,156 respondents (mean age: 34.3 years; 83.1% female). The IAT had two factors: “impairment in time management” and “impairment in socio-emotional functioning” of which respondents had more impairment in time management than socio-emotional functioning. Based on the original IAT, 19.4% of respondents would be classified as having a mild internet use problem with 3.0% having a moderate or severe issue. In the multivariable model, perceived stress (b = .28, SE = .05, p < .001) and depressive symptoms (b = .24, SE = .10, p = .017) were associated with higher IAT scores. Handheld mobile devices were the most common type of screen used (mean = 3 hours/day) followed by watching television (mean = 2 hours/day).

**Conclusion:**

Parents spent a significant portion of their time each day using screens, particularly handheld mobile devices. The disruption caused by mobile devices may hinder opportunities for positive parent-child interactions, demonstrating the need for resources to support parents ever-growing use of technologies.

## Introduction

In the past decade, there have been profound changes in the types of digital technologies available to the general population, with a particular increase in the use of mobile devices in the home [1]. Near ubiquitous access to these devices has changed individuals’ daily exposure to screen time in duration (e.g., time spent using devices) and content (e.g., social media, news, television, movies). In 2016, 94% of Canadians 15–34 years owned a smartphone and habits established in this time likely persist throughout adulthood [2]. Traditional screen time exposure, meaning “time spent with the television on”, is associated with fewer verbal exchanges between parents and children [3], decreased initiated interactions [4], and decreased language-enriching activities [5].

Recently, a new wave of research has examined these associations with parent mobile device and internet use. Mobile device use is associated with a decrease in verbal and nonverbal interactions and encouragement [6]. The disruption in parent-child interaction due to technology has even been labeled “technoference” [7]. In one longitudinal study, parents’ interrupted attention by their devices was associated with child externalizing behaviors and parenting stress [8]. These trends are concerning, but current research is limited by small sample sizes and heterogeneous methods. As screen time behavior evolves from families having one or two television sets per household to each member having two or more handheld devices, new measures are required to capture these exposures. Understanding the patterns of screen use and burden of problematic internet use in parents would inform resources to support parents navigate using technologies.

Several tools have been developed to measure problematic internet use. The most popular is Young’s Internet Addictions Test (IAT) [9], a 20-item scale on screen use and internet behaviors. A systematic review demonstrated that the IAT has good psychometric properties [10]. However, the factorial structure is inconsistent across studies with one to five factors being reported often with problematic cross-loadings, indicating unclear factor separation. While the review authors suggested the correct solution likely has one or two factors [10], this conclusion was mostly based on samples of university students in Asia or Europe. The internal consistency and construct validity of the IAT for use in parents in a North American context needs to be established to support research in this area. Therefore, our objectives were to: (1) establish the factorial structure and internal consistency of the IAT in a sample of parents (Study 1), and, with its validity and reliability being supported, (2a) establish the level and correlates of problematic internet use in parents and (2b) report on their patterns and types of screen use (Study 2).

## Methods

### Sample

Data were derived from a survey of preconception care attitudes, beliefs, and intervention preferences of women and men in across Canada, undertaken in May to June, 2019. Participants were recruited via advertisements on public health unit websites, online study promotion on parenting webpages, identification of eligible individuals through existing research datasets, and referrals from ongoing studies. Women and men were eligible if they could read and understand English, were able to access a telephone or the Internet, and for the current analyses, had ≥1 child in the past 5 years and provided data on internet and screen use. Individuals interested in participating received an introductory email after contacting the research team. Those who were eligible and agreed to participate received a link to an online consent form and questionnaire using the Research Electronic Data Capture (REDCap) system. Research staff assisted those who had difficulty accessing the online questionnaire and sent reminder follow-up telephone calls. The study received ethics approval from the University of Toronto and the University of Alberta.

### Measures

#### Internet and screen use

Internet and screen use were assessed using the IAT [9], a self-report measure that contains 20 items rated using a Likert-scale ranging from *not applicable* (0) to *always* (5). The total score is calculated by summing item responses (range: 0 to 100), with higher scores indicating a higher severity of internet disorder. Young [9] reported cut-offs for categorizing internet behavior into four levels of impairment: no (0–30), mild (31–49), moderate (50–79), or severe (80–100). We also collected data on the presence and number of televisions, DVD/video players, computers/laptops, video game consoles, and handheld devices (e.g., iPhones, tablets, Nintendo DS videogames) in the home and screen time according to device type for weekdays and weekends (“On a typical day, how many hours did you spend: watching television including streaming; watching videos/DVDs; using the computer/laptop (not for work); playing video games; using handheld devices?”). To ensure validity, we removed impossible values of > 24 hour of screen use per device/per day. Questions were asked about the presence of a television in the bedroom (*yes*/*no*) and screen use while eating (“On a typical day, which meals do you eat with a screen device on?” *yes*/*no* for breakfast, lunch, dinner, and snack on weekdays and weekends). Finally, two *yes*/*no* questions were asked about overuse: “Do you think you use your screen devices too much?” and “Would you like to decrease the amount of time you spend on a screen device?” (for the latter question, indicating what devices they would like to use less).

#### Potential correlates of internet and screen use

The questionnaire contained questions on potential correlates of internet and screen use as identified in the literature: socio-demographics, mental health, health behaviors, and general health. Socio-demographic questions included age, sex, marital status, education level, income level, number of children, employment status, and province (or when sample size was too small, region). Mental health indicators were measures of depression (9-item Patient Health Questionnaire [PHQ-9] [11], assessing symptoms in the past 2 weeks; Cronbach’s alpha: .82 in the current sample), anxiety (7-item Generalized Anxiety Disorder scale [GAD-7] [12], assessing symptoms in the past 2 weeks; Cronbach’s alpha: .87), and stress (10-item Perceived Stress Scale [PSS] [13], assessing feelings in the past month; Cronbach’s alpha: .89). Health behaviors were: any smoking (“On a typical day, how many cigarettes do you smoke?” *none*/*any*), alcohol use (“How often do you drink a beverage containing alcohol?” *daily or almost daily/other*), regular (non-medicinal) cannabis use (“In the past 12 months, have you used cannabis [marijuana] for non-medical/recreational reasons?” followed by frequency of use, *regular users*/*other*), and physical activity level (Global Physical Activity Questionnaire [GPAQ], [14] assessing total physical activity MET-minutes/week, with those scoring <600 coded as not meeting World Health Organization physical activity level standards). Finally, general health was assessed using the question: “How would you rate your overall health?” (*very healthy* (1), *healthy* (2), okay (3), *unhealthy* (4), and *very unhealthy* (5)).

### Statistical analyses

#### Study 1: Establishing the factorial structure of the IAT among parents

The sample for Study 1 consisted of 1,156 participants who had ≥1 child and responded to the IAT. The sample was split randomly into two halves, to do an Explorative Factor Analysis (EFA) in one half (n = 580) and a Confirmatory Factor Analysis (CFA) in the other (n = 576), using Mplus (v. 7). The literature is inconsistent as to whether the scale should be analyzed using Pearson’s correlations or polychoric correlations, which account for the ordinal response options, so we tested both methods, the former with a robust maximum likelihood estimator and the latter with a weighted least squares estimator. Factors were extracted using parallel analysis and Velicer’s Minimum Average Partial (MAP) test in SAS [15]. Mplus’ default rotation method of GEOMIN was tested, as well as OBLIMIN to allow for correlations between factors to find a solution with the best factor separation. The factorial structure that was indicated by the EFA was then tested in the other half of the sample using CFA using the same correlation type and estimator as the final EFA model. Model fit was assessed using the Root Mean Square Error of Approximation (RMSEA, <0.06 recommended), Comparative Fit Index (CFI, >0.95 recommended), and the Tucker-Lewis Index (TLI, <0.95 recommended). Modification indices were requested to explore sources of model misfit. Once the factorial structure was confirmed using CFA, composite reliability was calculated for each subscale based on the standardized factor loadings and error variances [16].

#### Study 2: Measuring screen use and internet addiction among parents

To be consistent with Study 1, the sample for Study 2 included parents who responded to the IAT (n = 1,156). IAT mean total and subscale scores were calculated, with their standard deviations (SD) and the frequency and percentage of participants who fell into each of Young’s 4 categories [9]. This was performed for the overall sample and for women and men separately. An independent t-test was used to compare total score between women and men. Correlates of IAT scores were assessed using multivariable linear regression. Model diagnostics, including inspecting the distribution of residuals, testing for multicollinearity, and confirming linear relationships between continuous predictors (e.g., age) and the outcome, were undertaken before selecting a final model. All variables that were determined *a priori* as potential correlates were left in the final model regardless of significance. The mean, SD, median, and interquartile range (IQR) were calculated for daily screen use time by device type for weekdays and weekends separately. Using the weekday and weekend data, we calculated a weighted mean to determine average screen time per device. We also recorded the frequency and percentage of number of devices used in the household, screen use at meals, and positive responses to questions about using screens too much and wanting to reduce screen time. Finally, differences between those with a potential internet use problem (mild/moderate/severe) according to the IAT and those without a problem were tested according to: duration of screen time use (independent t-tests), use of screens during meals (Chi square test), and perception of overuse and desire to decrease use of screens (Chi square tests). Statistical significance was set at < .05. The latter analyses used SAS (version 9).

## Results

In total, 1,265 parents responded to the questionnaire of which 1,156 (961 women, 195 men) completed the IAT and were included in the final sample. Demographic characteristics of the analytic sample are provided in Table 1. The respondents had a mean age of 34.3 (SD = 4.5), 83.1% were women and 95.6% were married. Three-quarters (76.6%) had a university degree, 64.5% were currently working for pay, and 30.4% had a household income between $100,000 and $149,000. About a half of respondents had two children (51.4%) and 35.1% had one only. Most respondents were living in Ontario, the most populous province in Canada (65.0%). When comparing those in the analytic sample to parents without data on the IAT, a higher proportion of those with missing IAT data had lower education (p = .041) and lower income (p = .028).

**Table 1:** Demographic characteristics of the sample (n = 1156).

	N (%) or Mean (sd)
**Age**	34.3 (4.5)
**Sex**	
Women	961 (83.1)
Men	195 (16.9)
**Marital Status**	
Married/Common-Law	1105 (95.6)
Single/Divorced/Widowed	51 (4.4)
**Education Level Completed**	
High school or college	270 (23.4)
University	886 (76.6)
**Employment Status**	
Work for pay	746 (64.5)
Unemployed, on leave, in education or other	410 (35.5)
**Household income**	
<$25,000	42 (3.6)
$25–49,000	108 (9.3)
$50–74,900	178 (15.4)
$75–99,000	216 (18.7)
$100–149,000	351 (30.4)
$150–199,000	164 (14.2)
$200–299,000	77 (6.7)
$300,000 +	20 (1.7)
**Number of children**	
One	406 (35.1)
Two	594(51.4)
Three or more	156 (13.5)
**Geographic Region**	
British Colombia & Yukon	129 (11.1)
Alberta	111 (9.6)
Prairies (Manitoba and Saskatchewan)	65 (5.6)
Ontario	758 (65.0)
Quebec	36 (3.1)
Eastern Coast (Nova Scotia, New Brunswick, Newfoundland)	57 (4.9)

### Study 1. Establishing the factorial structure of the IAT among parents

#### Exploratory factor analysis

Parallel analysis indicated the extraction of two factors based on parallel analysis and the MAP test, explaining 58.1% of the total variance (Table 2). The best model based on factor separation and strength of factor loadings was specified using the polychoric correlations, weighted least square estimator and the GEOMIN rotation. The first factor corresponded with the concept of “impairment in time management” and included items 1, 2, 3, and 7. The second factor included the remaining items and aligned with the concept of “impairment in socio-emotional functioning”. Standardized loadings in Factor 1 ranged from .78 to .33 and in Factor 2 from .98 to .38. Five items, however, exhibited problematic cross-loadings: 3, 6, 8, 16, and 17.

**Table 2:** Standardized factor loadings from an exploratory factor analysis (n = 580).

	Item	Factor Loadings	
		1	2
1	How often do you find that you stay online longer than you intended?	**0.784**	‒0.02
2	How often do you neglect household chores to spend more time online?	**0.683**	0.132
7	How often do you check your e-mail before something else that you need to do?	**0.368**	0.201
3	How often do you prefer the excitement of the Internet to intimacy with your partner?*	**0.331**	0.331
20	How often do you feel depressed, moody, or nervous when you are off-line, which goes away once you are back online?	‒0.18	**0.981**
19	How often do you choose to spend more time online over going out with others?	‒0.114	**0.895**
15	How often do you feel preoccupied with the Internet when off-line, or fantasize about being online?	0.01	**0.887**
18	How often do you try to hide how long you’ve been online?	‒0.005	**0.829**
12	How often do you fear that life without the Internet would be boring, empty, and joyless?	‒0.073	**0.777**
11	How often do you find yourself anticipating when you will go online again?	0.118	**0.763**
9	How often do you become defensive or secretive when anyone asks you what you do online?	0.011	**0.754**
10	How often do you block out disturbing thoughts about your life with soothing thoughts of the Internet?	‒0.004	**0.738**
13	How often do you snap, yell, or act annoyed if someone bothers you while you are online?	0.097	**0.734**
16	How often do you find yourself saying "just a few more minutes" when online?*	0.413	**0.517**
14	How often do you lose sleep due to late-night log-ins?	0.325	**0.506**
4	How often do you form new relationships with fellow online users?	0.068	**0.492**
17	How often do you try to cut down the amount of time you spend online and fail?*	0.397	**0.453**
5	How often do others in your life complain to you about the amount of time you spend online?	0.239	**0.44**
6	How often does your work, grades or school work suffer because of the amount of time you spend online?*	0.355	**0.439**
8	How often does your job performance or productivity suffer because of the Internet?*	0.349	**0.382**

*Items were removed due to double cross-loading.

#### Confirmatory factor analysis

The two-factor model was tested using CFA in the second half of the sample, with the 5 items involved with problematic cross-loadings removed. Two of these items (8 and 17) have been removed in other studies for the same reason [17]. The model fit the data well according to the CFI (.98) and TLI (.97), and had slightly less than adequate fit according to the RMSEA (.08). An inspection of modification indices suggested that correlating the error variances of some of the items may improve model fit, which has also been shown in other studies [18]. However, we did not make any further modifications. For the purposes of providing a valid tool for Study 2, we assessed internal consistency of the two subscales and the overall 16-item scale by calculating composite reliability; these were .72 (Factor 1), .94 (Factor 2), and .95 (overall 15-item scale). With confirmation of the factorial structure and internal consistency of the IAT among parents, we moved to the second study.

### Study 2. Measuring screen use and internet addiction among parents

#### IAT scores

IAT scores for the original 20-item scale ranged from 0 to 84, with a mean of 23.5 (SD = 11.5) out of a possible total of 100; for the revised 15-item scale, scores ranged from 0 to 63, with a mean of 17.4 (SD = 8.6) out of a possible total of 75 (Table 3). The time management impairment subscale mean was 6.4 (SD = 2.5) out of a possible total of 15, and the socio-emotional functioning impairment subscale mean was 11.0 (SD = 7.0) out of a possible total of 60. When looking at the mean as a proportion of the highest possible total score, respondents had more impairment in time management than socio-emotional functioning. Separating women and men, women had significantly higher scores for the original scale, the revised scale, and each subscale. According to the categories proposed by Young [9], based on the full 20-item scale, 77.7% (n = 898) would not be considered to have a problem, 19.4% (n = 224) would have a mild problem, and 3.0% (n = 34) would have a moderate or severe problem. For the stratified analysis, we grouped mild, moderate and severe into one category based on sample size. For women, 729 (75.9%) had no problem and 232 (24.1%) had a mild/moderate/severe problem. For men, 169 had no problem (86.7%) and 26 (13.3%) had a mild/moderate problem (none were severe).

**Table 3:** Mean Internet addiction test scores between women and men.

	Full Sample (n = 1156)		Women (n = 961)		Men (n = 195)		*t* *	p
	Mean	SD	Mean	SD	Mean	SD		
IAT Total—Revised 15 item scale	17.37	8.58	17.79	8.71	15.30	7.61	4.06	< .001
IAT Total—Original 20 item scale	18.15	9.03	24.08	11.72	20.67	10.07	3.79	< .001
IAT subscale—"time management impairment"	6.38	2.51	12.03	7.60	10.52	6.71	2.80	0.01
IAT subscale—"socio-emotional impairment"	11.00	7.05	11.23	7.16	9.80	6.32	2.82	0.005

*independent t-test comparing mean scores for women and men.

#### Correlates of IAT scores from multivariable regression

Multivariable linear regression was used to identify correlates of the IAT score (n = 1,153) based on the revised 15-item scale: (1) a model predicting the total score, (2) a model predicting the time management impairment score, and (3) a model predicting the socio-emotional impairment score (Table 4). Note that the models should be compared with caution as the score ranges vary between subscales. In Model 1, older age (b = -.15, SE = .06, p = .007) and any smoking (b = -2.32, SE = 1.17, p = .048) were associated with lower scores, while perceived stress (b = .28, SE = .05, p < .001) and depressive symptoms (b = .24, SE = .10, p = .017) were associated with higher scores. In Model 2, male gender was associated with lower scores (b = -.77, SE = .21, p < .001), while higher household income (b = .15, SE = .05, p = .003) and more perceived stress (b = .05, SE = .03, p = .002) were associated with higher scores. In Model 3, older age (b = -.13, SE = .05, p = .007) was associated with lower scores, while perceived stress (b = .23, SE = .04, p < .001) and depressive symptoms (b = .20, SE = .08, p = .016) were associated with higher scores.

**Table 4:** Correlates of the Internet addiction test score and subscale scores from multiple linear regression (n = 1153).

	Total score (15 items)			Time-Management Subscale			Social-Emotional Subscale		
	B	Std. Error	p	B	Std. Error	p	B	Std. Error	p
Male sex	‒0.87	0.68	0.198	‒0.77	0.20	**<0.001**	‒0.09	0.56	0.866
Working for pay (ref = unemployed, on leave or in education)	0.05	0.54	0.728	0.03	0.16	0.830	0.01	0.44	0.984
University graduate (ref = high school or college grad)	0.42	0.60	0.489	0.15	0.18	0.397	0.27	0.50	0.584
Two or more children (ref = 1)	0.61	0.51	0.232	0.11	0.15	0.468	0.49	0.42	0.242
Household income	0.05	0.17	0.771	0.15	0.05	**0.003**	‒0.10	0.14	0.483
Age	‒0.15	0.06	**0.007**	‒0.03	0.02	0.108	‒0.13	0.05	**0.007**
Perceived stress (PSS)	0.28	0.05	**<0.001**	0.05	0.02	**0.002**	0.23	0.04	**<0.001**
Depression (PHQ-9)	0.24	0.10	**0.017**	0.04	0.03	0.177	0.20	0.08	**0.016**
Anxiety (GAD-7)	0.06	0.10	0.519	0.04	0.03	0.198	0.03	0.08	0.752
Self-rated health	0.05	0.39	0.891	0.16	0.12	0.170	‒0.10	0.32	0.751
Weekly or daily cannabis use	‒2.11	1.44	0.142	‒0.72	0.43	0.093	‒1.36	1.19	0.251
Any smoking	‒2.32	1.17	**0.048**	‒0.61	0.35	0.078	‒1.78	0.97	0.067
Daily alcohol use	0.15	1.10	0.900	‒0.09	0.33	0.772	0.25	0.91	0.781
Physically active	‒0.86	0.50	0.086	‒0.11	0.15	0.454	‒0.74	0.41	0.074

#### Screen use duration

**Fig 1:**
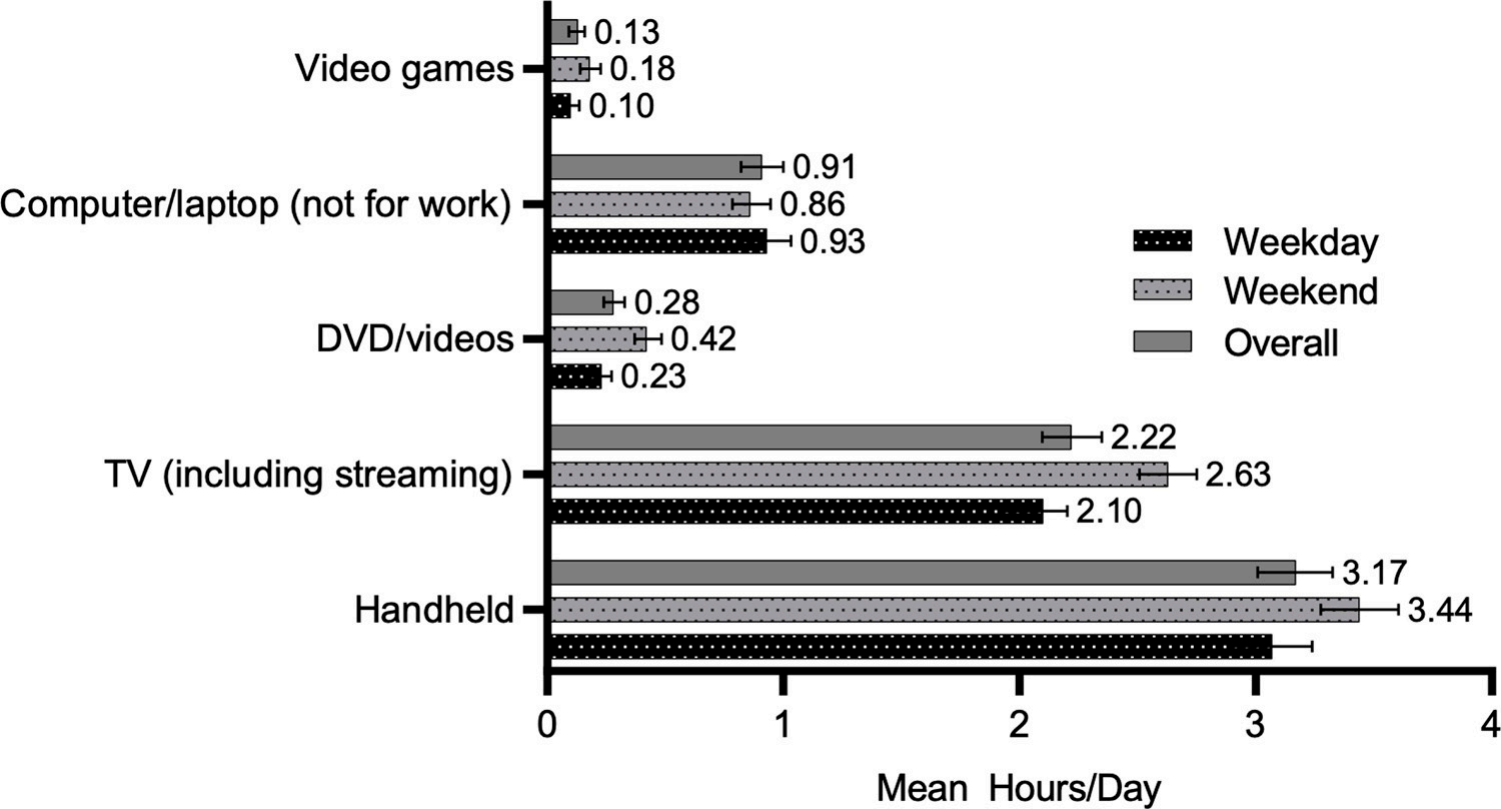


For an average weekday, most time was spent using handheld devices, with a mean of 3.07 (SD = 3.0) hours/day (median = 2, IQR = 1–4), followed by watching television (mean = 2.1, SD = 1.5; median = 1.5, IQR = 1–2) and using a computer for non-work purposes (mean = .93, SD = 1.8; median = 0, IQR = 0–1). Mean screen time for weekdays was low for watching videos/DVDs or playing video games. On the weekend, more time was spent using screens. Most time was spent using handheld devices, with a mean of 3.44 (SD = 2.84) hours/day (median = 3, IQR = 2–4), followed by watching television (mean = 2.63, SD = 2.10; median = 2, IQR = 1–3) and using a computer for non-work purposes (mean = .86, SD = 1.41; median = 0, IQR = 0–1). Mean screen time for weekend days was low for watching videos/DVDs and playing video games. See Fig 1. When comparing average duration of screen use by device between parents with no problem according to the IAT and those with a mild/moderate/severe problem, the latter had significantly longer duration of screen use for all device types excluding video game consoles (handheld: p < .001; TV: p = .024; DVD: p < .001; video game: p = .642; computer: p < .001). For handheld device, those with no problem had a mean of 2.90 hours/day (SD = 2.5), compared to 3.75 (SD = 2.79) for a mild problem and 6.34 (SD = 5.18) for a moderate problem.

#### Devices in the home

Among respondents (n = 1,156), 95.2% reported having a television in their home, with 52.2% reporting ≥2 televisions and 31.7% indicating they had one in their bedroom. Nearly all respondents reported having a computer/laptop in their home (96.1%), with almost 63.4% reporting ≥2. Just under two-thirds indicated having a video game console (61.5%) or DVD/video player (64.0%), with 26.7% and 15.4% reporting ≥2, respectively. Handheld devices were reported in nearly all homes (96.5%), with 89.5% having ≥2, and 34.6% having ≥4.

#### Screen use at meals

At least one-quarter of respondents reported having a screen on during ≥1 weekday meal: 27.4% at breakfast, 37.8% at lunch, 24.8% at dinner, and 64.5% for snacks. These figures are similar on weekends, although screen use during lunch was lower: 29.2% at breakfast, 23.4% at lunch, 24.0% at dinner, and 59.9% for snacks. A higher proportion of parents with a potential problem based on IAT scores reported screen use during each type of meal on weekdays and weekends compared to those without a problem; breakfast (36.4% vs. 24.8% on weekdays and 38.0% vs. 26.7% on weekends; both p < .001), lunch (47.7% vs. 35.0% and 32.6% vs. 20.3%; both p < .001), dinner (33.3% vs. 22.4%, p < .001 and 29.8% vs. 22.3%, p = .01), and snacks (74.4% vs. 61.7%, p < .001 and 69.0% vs. 57.3%, p = .001).

#### Perceptions on overuse

When asked “Do you think you use your screen devices too much?”, 72.7% responded “yes” and 76.0% reported they would like to decrease the amount of time spent on a screen device. The most common devices for which reduced screen time was desired were hand-held devices (73.6%), televisions (17.3%), and computers (6.3%). When comparing perceptions of overuse between parents with no problem according to the IAT and those with a mild/moderate/severe problem, a significantly higher proportion of latter thought that they used screen devices too much (93.8% vs. 66.6%, p < .001). Similarly, a significantly higher proportion of parents with a potential problem (90.3%) than those without a problem (73.5%) indicated that they wanted to reduce the amount of time spend on a screen (p < .001).

## Discussion

In this large Canadian study examining problematic internet use in parents, we found evidence that the IAT is comprised of two subscales: “impairment in time management” and “impairment in socio-emotional functioning”. Our data showed that over one in five parents had a mild to severe internet addiction, and mothers had higher rates of potentially problematic internet use than fathers, especially in the domain of time management. Perceived stress and depressive symptoms were associated with higher IAT scores. More time was spent using handheld devices rather than watching television. More than one in four parents were eating with screens during at least one meal per day, and parents with a potential internet use problem used screens at meals more frequently. A high proportion of respondents perceived their screen devices were used too much and had a strong desire to reduce screen time. Collectively, these findings have implications for the development of resources to support parents in appropriate screen use, to minimize negative impacts on child development.

Similar to current patterns of screen use in high-income countries [1], handheld devices were the most frequently used. The number of handheld devices in every home, and those with two or more devices, was also similar to national Canadian survey data [2]. Perceived stress was the only factor associated with total and subscale IAT scores, corroborating prior studies showing that screen use was both a stress-inducing and a stress-relieving necessity in families [8]. For example, in one qualitative study parents described multitasking between technology use and children as stressful or less effective because their attention was divided and it was difficult to read and respond to children’s social cues [8]. Mobile devices provided stress-relieving conditions for parents to withdraw, but also led to displacing opportunities for positive parent-child interactions [8]. Other studies reported parents’ experiences of internet and mobile device use as rewarding when they were able to disengage from family and the boredom of child-rearing [19, 20]. Although we found positive correlations between depressive symptoms and higher IAT scores, there is mixed evidence in the literature on this association. For example, one study found no associations with maternal depression [6], while another found smartphone interruptions were associated with higher maternal depressive symptoms [7]. Finally, we showed that the proportion of parents reporting perceived overuse of screen devices was quite high, consistent with prior studies showing that 40% of parents wanted to decrease their screen use [21].

A main concern of excess screen use by parents is the potential disruption of parent-child interactions, particularly for young children [22]. It is well-established that quality parent-child interactions are the foundation to support healthy development by encouraging serve-and-return, parental responsiveness, and sensitivity [23]. Parents constantly connected to their mobile devices may disrupt opportunities for these important developmental processes. In particular, the function of handheld devices and the persuasive design of social media applications [24] encourages increased screen time. Understanding some of these patterns may help to guide parents to ways of reducing the potential harms of screen use. One mixed methods study found all parents interviewed believed their device use was affecting their parenting [19]. Another concern for parents’ problematic internet and mobile device use is the increase in a child’s own screen use [25], which may be associated with poor health outcomes. Very few studies have examined the relationship between children’s own exposure to mobile devices and health outcomes. Research in this area is still evolving as methods to accurately capture screen time and content are further developed. A study of children’s early-life screen exposure and health behaviours, such as 24-hour movement behaviors (e.g., physical activity), showed screen use was detrimental to a child’s physical movement [26]. A systematic review determined increased screen time was associated with poor sleep outcomes in children under 5 years [27]. One study found screen time duration, including mobile devices, was associated with expressive language delay in 18-month-old children [28]. It is notable that parents with potential internet use problems had increased meals with screens. Children’s screen use at mealtime is associated with poor eating behaviours, increased unhealthy and highly advertised food intake, and decreased fruit and vegetable intake [29, 30]. A recent systematic review and meta-analysis including 20 observational studies (n = 84,825) identified a positive association between television viewing during mealtime and risk of overweight/obesity in children <18 years [29]. Increased internet use and mealtime screen use by parents may be an important risk factor related to health outcomes in both adults and children.

Strengths of our study include our large sample from across Canada, including fathers, who represented approximately 20% of the sample. Establishing the factorial structure of the IAT and confirming this analysis in half of the study sample demonstrated the validity and reliability of this scale for the study objectives. These novel and formative data on parent internet addiction and screen use will support future research in how screens can affect the parent-child relationship and how that may be addressed in the preconception period. However, although a large number of Canadians responded to our survey, this study captures data from those who elected to respond. Of note, five mothers responded to the survey for every one father, suggesting that the latter may comprise a more selected subpopulation. Fathers more willing to participate in surveys about preconception care may be more committed to parenting and therefore more mindful of their screen usage. Further, our sample was of a relatively high socioeconomic status, and most respondents were married. Relatedly, those with low education and low income were more likely to have missing IAT data. Future research using representative sample is needed before strong conclusions about internet addiction in Canadian parents can be established. Data on screen use duration was self-reported and there was no way of knowing if multiple devices were being used concurrently. Moreover, screen use dedicated to child education could not be disentangled from overall screen use and while we would assume this type of usage would be minimal given the young age of the children of the sample parents, more objective methods for collecting screen use data should be included in future research. Finally, this was also a cross-sectional study; therefore, causation cannot be inferred.

This study provides evidence to understand current patterns of problematic internet and screen use by parents. Future research is needed to understand the relationship between parents’ screen use and child and parent health behaviours and outcomes. Additionally, examining the content and context of use, including mealtime use, by parents and their children may inform the mechanisms of poor child and family outcomes. Health care providers, public health practitioners, and policy makers should support increased public awareness of how screens may affect familial relationships and child development.

## Supporting information

S1 Data (XLS)

S1 File Request for change to authorship. (DOCX)
